# Tailoring Weight Loss Before Hernia Surgery: Distinguishing Between Two Types of Obesity

**DOI:** 10.3389/jaws.2025.14322

**Published:** 2025-03-12

**Authors:** John M. Findlay, David L. Sanders

**Affiliations:** ^1^ Academic Department of Abdominal Wall Surgery, North Devon District Hospital, Royal Devon University Healthcare NHS Foundation Trust, Barnstaple, United Kingdom; ^2^ Department of Clinical and Biomedical Sciences, University of Exeter Medical School, Exeter, United Kingdom; ^3^ NIHR Exeter Biomedical Research Centre, Exeter, United Kingdom

**Keywords:** hernia, obesity, weight loss, prehabilitation, outcomes

## Introduction

Complication and recurrence rates following hernia repair remain unacceptably high, especially in larger and more complex cases, where rates can exceed 25% [1]. While there is some degree of consensus on certain technical aspects of the surgery—such as approach, mesh placement, overlap, and mesh type—the specifics of preoperative risk reduction remain less clear. This is especially true beyond the overarching concept of “prehabilitation,” which involves optimizing patients’ health prior to surgery by addressing modifiable risk factors [1].

Key preoperative risk factors for recurrence and wound complications after hernia surgery include body weight, smoking, and diabetic control [1]. Ventral and incisional hernia surgery is usually elective and considered “quality-of-life” surgery, providing an opportunity to optimize patients’ health before the procedure. In the United Kingdom, the “Getting it Right First Time” (GIRFT) initiative advocates for preoperative optimization and even suggests delaying surgery if necessary to improve patient outcomes [2].

In this paper, we outline the challenges of managing obesity in patients with incisional or primary ventral hernias and provide a novel practical guide for differentiating patients into two groups. This classification aims to guide the approach to pre-optimization, a strategy not previously described in the literature.

## The Challenge of Weight Loss

One of the most significant challenges in preoperative optimization is modifying body weight. A large proportion of patients undergoing ventral hernia repair are obese, which substantially increases the risk of complications and recurrence—risks that are even more pronounced in complex cases such as large incisional or recurrent hernias [3, 4].

Visceral fat, in particular, is thought to predict hernia recurrence due to increased biomechanical stress, while subcutaneous fat is associated with postoperative complications, particularly surgical site occurrences, which may influence recurrence rates [5]. Furthermore, obesity is a major metabolic risk factor that can negatively impact surgical outcomes [6].

The presence of a hernia—especially a large one—can make weight loss more challenging. This is particularly true when other modifiable risk factors, such as smoking, are also being addressed. Both functional and psychological factors complicate weight loss efforts, and as a result, many patients fail to achieve significant weight reduction with general weight-loss advice alone [3].

Medications such as GLP-1 analogues have shown dramatic results in large-scale studies, particularly in patients with Type 2 diabetes, who face additional challenges in managing weight and blood sugar [7, 8]. While these drugs have not yet been studied in the context of prehabilitation before surgery, their potential to optimize patients preoperatively is promising.

While weight loss is generally recommended before surgery [1], the optimal method (e.g., calorie restriction, exercise, pharmacological intervention) and how to set an appropriate target weight remain unclear. Delaying surgery in hopes of achieving weight loss requires a delicate balance, as there is a risk that the hernia could become more complex or even irreparable during the waiting period.

## Personalizing Weight Loss Before Surgery

We propose that preoperative weight loss should be personalized based on several factors, most importantly:

• Clinical Urgency
• Technical Complexity
• Practicality

### Clinical Urgency

In some cases, the urgency of hernia repair may make attempts at weight loss inappropriate. However, for most patients the risk of an emergency complication requiring urgent surgery without attempts at risk reduction through pre-optimisation is low. Conditions such as recurrent emergency presentations with severe pain or obstructive symptoms which resolve with non-operative management, as well as certain radiological features such as a high hernia-to-defect ratio (a large hernia through a small defect) and acute angulation of the hernia sac relative to the abdominal wall (as seen on CT scans), may suggest the need for urgent repair [9, 10]. Other factors include rapidly enlarging hernias which risk becoming more complex to repair (or even irreparable) with delay. In the absence of reliable tools by which to predict hernia progression and complications, clinical urgency remains subjective and imprecise, and as with the other factors below should be incorporated into a collaborative discussion of risk and benefit with patients.

### Technical Complexity

More complex hernias, especially those with large fascial defects and significant muscle atrophy, carry a higher risk of complications. These cases are more likely to require myofascial release to achieve closure, which itself carries risk. In this group, weight loss may be beneficial to reduce visceral fat and, consequently, the volume of intra-abdominal contents. However, the time required for weight loss could inadvertently result in the hernia enlarging, or lead to further muscle atrophy, thereby increasing the complexity of the eventual repair. This is particularly concerning for patients with recurrent hernias or those requiring advanced techniques like myofascial release, and those at risk of loss of domain [11, 12].

### Practicality

The practical feasibility of weight loss varies among patients. Some may have previously attempted and failed to lose weight, while others may have already achieved significant weight loss, leaving limited potential for further reductions. From a healthcare systems perspective, balancing the availability of resources and time is crucial. Delaying surgery for ultimately futile or ineffective attempts at weight loss may increase strain on already limited surgical slots and resources, and this may become apparent during a trial of pre-habilitation.

## Two Types of Weight Loss Required in Hernia Surgery

**FIGURE 1:**
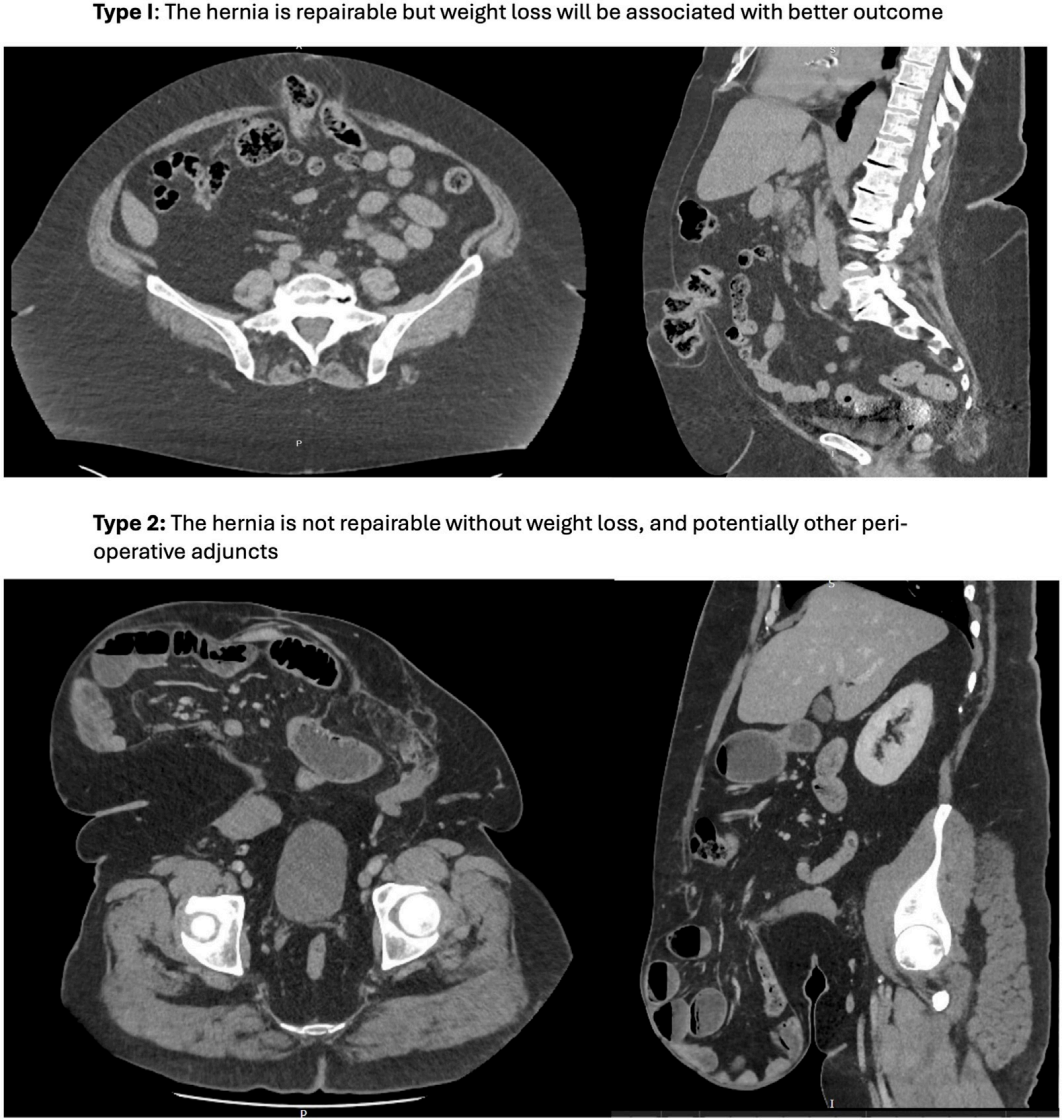
Two types of obesity in patients with the same BMI (49). Type I: The hernia is repairable but weight loss will be associated with better outcome. Type 2: The hernia is not repairable without weight loss, and potentially other peri-operative adjuncts.

Managing obesity before hernia surgery is therefore complex, with a number of often competing issues. To help navigate this, we find it helpful to differentiate between two types of preoperative obesity. These are illustrated in Figure 1, which demonstrate incisional hernias in patients with the same BMI (49), but different types of obesity.

1. Type I: For most patients, weight loss is preferable but not mandatory. Surgery is feasible at the current weight, but the risk of complications and recurrence may be reduced with weight loss. The target weight loss is individualized and dynamic, taking into account clinical urgency, technical complexity, and practicality, in addition to the presence of other risk factors such as diabetes which may compound risk.
2. Type II: In some cases, the patient’s weight and fat distribution make surgery technically unfeasible without weight loss, often due to loss of domain. For these patients, weight loss is not just beneficial; it is essential for making surgery possible.

Pre-operative weight loss can therefore be considered a perioperative adjunct to make a hernia repairable, such as chemical component separation with botulinum toxin, peritoneal flap hernioplasty, myofascial release (anterior component separation and transversus abdominis release) and progressive pneumoperitoneum. However, these may not be options for all patients (either due to local resources, or patient-specific factors), and their success and ability to utilise them may be unpredictable—for example, an incisional hernia in the presence of a laparostomy scar or mesh to be excised potentially precluding a peritoneal flap hernioplasty; or chemical component separation in a patient with broad and non-retracted lateral muscle complexes. By contrast (in principle) weight loss is achievable and its effects can be assessed before surgery using CT imaging.

## Using This Classification in Multidisciplinary Practice

Our experience suggests that this classification of obesity types is a valuable tool for guiding collaborative decision-making with patients and multidisciplinary teams, as well as aiding the pre-optimisation process itself. For patients with Type I obesity, weight loss is strongly encouraged, but in principle surgery can proceed even if the target weight loss is not fully achieved (or achieved at all). The focus in these cases is on minimizing the risk of complications and recurrence by optimizing weight to a clinically significant degree. In addition, recognition of the impact of type 1 obesity on complications can help guide technical decisions; for example, considering techniques with lower wound complication risks such as a minimally invasive approach rather than open.

What this means in practice for individual patients has to be personalised, taking into account clinical urgency, technical complexity, the degree of obesity and synergy with risk factors, and what is practically achievable for patients and healthcare systems, rather than stipulating arbitrary and “one size fits all” targets. For example, for patients with type 1 obesity but at a relatively low BMI (e.g., 31), with a largely subcutaneous adipose distribution, with a relatively small hernia (perhaps repairable with a minimally invasive approach without additional techniques such as transversus abdominis release, thereby minimising wound complications), without concomitant risk factors, with significant episodes of pain suggesting a degree of urgency, and who have already lost considerable weight (perhaps plateauing following weight loss surgery), prolonged attempts at weight loss to reach an arbitrary 10% target may carry more risks through delay than benefits. We would explain the importance of weight loss, and encourage as much weight loss as possible before surgery, but not delay their surgery unnecessarily to achieve this.

By contrast, our approach would be different for a patient with type I obesity at a BMI of 45, with a large and complex hernia which will require an open approach involving myofascial release (therefore conferring substantially higher wound risks), which shows little change over time, with no symptoms to suggest particular urgency, who also has diabetes (thereby increasing risks further), but has not previously tried to lose weight (and so has the potential to do so significantly). In these patients, we would set a guide target of perhaps 15%, and keep the patient under close review with ongoing support. If he or she can exceed this target then we may delay surgery further and extend the target; however, if a significant component of this risk benefit ratio were to change (such as an emergency presentation) then we may abort prehabilitation and proceed to surgery.

For patients with Type II obesity, weight loss is mandatory. Surgery cannot proceed without significant weight reduction, making it easier to focus prehabilitation efforts on achieving this goal. With close support, clinical and radiological review our goal is to covert type II obesity to type I obesity, and then re-evaluate ongoing weight loss as above.

## How to Distinguish Type I and II Obesity

The distinction between Type I and Type II obesity is based on whether the hernia is repairable at the patient’s current weight. However, this assessment can be influenced by several factors, including hernia anatomy, abdominal anatomy, fat distribution, prior surgeries, and patient risk factors [13–16]. This distinction is clear at the extremes but may become more nuanced in a small proportion of patients, requiring a comprehensive approach that draws on clinical experience and, particularly, up-to-date CT scans (including considering what other perioperative adjuncts are available to the surgeon and patient, and how successful they are likely to be).

At the risk of over-simplification, we recommend two complimentary tests of hernia volume relative to the abdominal volume, for the surgeon who is concerned: one radiological and one clinical. Whilst these are by no means definitive (and carry the risk of a false negatives and positive), they are useful when considered with all the other aspects discussed above.

### Radiological Assessment

The relationship of hernia volume to abdominal volume is key. The concept of “loss of domain” describes a hernia that is so large that simple reduction of its contents and primary fascial closure either cannot be achieved without additional reconstructive techniques or carries significant risks due to raised intra-abdominal pressure [11]. Loss of domain can be calculated using CT volumetry, typically employing the Sabbagh method, where the percentage loss of domain is calculated as hernia sac volume divided by total peritoneal volume (hernia sac volume + abdominal cavity volume). While there is no precise evidence for specific cut-off points, loss of domain is often evident when the sac volume exceeds 20%–30% of total peritoneal volume [11].

### Clinical: GRACE Test–GRavity Assisted Abdominal Cavity Evaluation

We complement the radiological assessment with a clinical test to assess hernia reduction using gravity and gentle manipulation. This method determines whether a hernia at risk of loss of domain can be reduced and maintained without discomfort or respiratory compromise. In this test, the patient is positioned supine or laterally, depending on the hernia location. Whilst some hernias may not be reducible due to adhesions or a narrow neck, if the hernia can be reduced and stays reduced without discomfort and the patient can breathe comfortably, the hernia is likely repairable (Type I). If not, significant weight loss is mandatory (Type II). Whilst reduction of a hernia is commonly practised by surgeons we do not believe has previously been described in the literature as a test or sign to assess whether further weight loss is required.

## Differential Fat Distribution

Fat distribution plays a crucial role in hernia repair. Visceral fat—comprising mesenteric, omental, and hepatic fat—is a significant component of both hernia sac volume and abdominal cavity volume. Although overall obesity is associated with both recurrence and complications in incisional hernia surgery [3, 4], visceral fat, in particular, is associated with an increased risk of recurrence [5]. Intuitively, visceral fat causes biomechanical strain on the hernia repair site, but it also contributes to negative cardiometabolic effects [8]. Fortunately, both visceral and subcutaneous fat can be reduced through weight loss via calorie restriction and exercise [12, 17, 18].

While the specific method of weight loss will vary by patient and healthcare system, reducing visceral fat can make previously irreparable hernias repairable. Additionally, fat distribution may influence the choice of surgical technique, particularly in Type I obesity. For example, patients with more subcutaneous fat are more likely to experience wound complications, which may make minimally invasive surgery preferable.

Visceral and subcutaneous fat parameters (and their ratio) can be calculated in a variety of ways from CT or MRI scans. Most simplistically, a single slice of the abdomen can be used at L3, and the depth of visceral obesity (from vertebra to rectus sheath) and subcutaneous fat measured, and compared [19]. Or, more complex manual volumetric measurements can be performed over multiple slices at multiple abdominal level [20].

## Discussion

This paper highlights the significant role that obesity plays in the outcomes of hernia surgery, particularly in terms of complications and recurrence rates. Obesity, especially visceral fat, is a known risk factor for poor surgical outcomes, and managing weight preoperatively is crucial for optimizing patient health. However, the approach to weight loss prior to surgery remains complex, as there is no one-size-fits-all solution. The optimal method for weight loss—whether through lifestyle changes, exercise, calorie restriction, or pharmacological interventions—is still unclear, and the timing of weight loss relative to surgery requires careful consideration.

This paper proposes a novel classification system that distinguishes two types of obesity in hernia patients: Type I and Type II. Type I obesity represents cases where weight loss is recommended but not essential for surgery, while Type II obesity involves cases where weight loss is mandatory for surgery to be feasible. This distinction is based on factors like hernia anatomy, fat distribution, and abdominal volume, with radiological and clinical assessments playing a key role in making this determination. In particular, we recommend considering radiological evaluation and a clinical test to assess the ability of the hernia to reduce with gravity and gentle manipulation (GRACE test).

This paper emphasizes the importance of personalizing preoperative optimization based on clinical urgency, technical complexity, and practicality. In Type I cases, weight loss should be encouraged to reduce the risk of complications, but surgery can still proceed even if the target weight is not fully achieved. In Type II cases, significant weight loss is essential before surgery can proceed. This classification system provides a useful framework for multidisciplinary teams to guide decision-making and improve patient outcomes.

In conclusion, this paper calls for more research into the timing, methods, and targets for preoperative weight loss, emphasizing the need to balance the benefits of weight reduction with the risks of delaying surgery. By considering the impact of obesity in terms of hernia complexity and clinical urgency, this approach could help refine preoperative strategies and inform future clinical guidelines.
